# Procalcitonin as an Early Marker of Colorectal Anastomotic Leakage in Postoperative Colorectal Cancer Patients: A Systematic Review and Meta-Analysis

**DOI:** 10.7759/cureus.70647

**Published:** 2024-10-01

**Authors:** Lucia Villegas-Coronado, Karla Villegas-Coronado, Alejandro Urrea-Quezada, Diana Villegas-Coronado

**Affiliations:** 1 Department of Surgery, Hospital General Regional No. 1, Instituto Mexicano del Seguro Social, Obregon, MEX; 2 Department of Geriatrics, Hospital General de Zona No. 89, Instituto Mexicano del Seguro Social, Guadalajara, MEX; 3 Department of Agriculture and Veterinary Medicine, University of Sonora, Hermosillo, MEX; 4 Department of Chemical and Biological Sciences, University of Sonora, Hermosillo, MEX

**Keywords:** biomarkers, colon, meta-analysis, pct, procalcitonin, rectum

## Abstract

Background

The timely identification of colorectal anastomotic leakage (CAL) is still a significant challenge, and identifying reliable markers is essential to minimize patient morbidity and mortality. While procalcitonin (PCT) has shown promise as a biomarker for CAL, its effectiveness must be specifically evaluated in colorectal cancer patients. This systematic review and meta-analysis sought to assess the mean differences in PCT levels between individuals with and without CAL who underwent colorectal surgery for colorectal cancer.

Methodology

A comprehensive search of the "PubMed," "Scopus," and "Web of Science" databases was carried out, covering studies published through April 2024. The objective was to identify studies examining PCT levels in colorectal cancer patients who underwent colorectal surgery, with a particular focus on the occurrence of CAL. Data on the mean of PCT levels in CAL and non-CAL patients were extracted from the selected studies. The mean differences in PCT levels were subsequently analyzed for each postoperative day (POD).

Results

Seventeen articles were selected for inclusion in this systematic review. The statistical analysis included five eligible articles that assessed PCT levels in groups exclusively involving patients with colorectal cancer. The findings showed no significant increase in PCT levels in CAL patients compared to non-CAL patients on any POD when a leave-one-out sensitivity analysis was performed to validate the results.

Conclusions

To date, PCT levels should not be regarded as early indicators of CAL after colorectal surgery in patients with colorectal cancer. Additional research is necessary to evaluate if PCT could be a dependable marker for CAL in this particular setting.

## Introduction and background

Anastomotic leakage in colorectal tissue (CAL) following colorectal surgery remains a critical issue, leading to increased morbidity and mortality [1]. This complication also serves as an important predictor of negative outcomes, particularly in patients who have undergone surgery aimed at curing colorectal cancer [2].

At present, various methods are employed to detect CAL, including endoscopy, contrast enemas, and computerized tomography scans [3]. Despite advancements in these diagnostic tools, identifying CAL remains difficult [4,5]. Many cases are only discovered in advanced stages, leading to a higher need for surgical intervention [6,7]. Therefore, timely identification of CAL is essential to reduce the adverse effects associated with this condition.

CAL is characterized by a disruption in the colorectal wall at the anastomosis site, leading to communication between the inside and outside of the lumen [8]. C-reactive protein (CRP) is one of the most extensively studied inflammatory markers for diagnosing CAL [9,10]. Its role as a diagnostic tool for CAL is well recognized. However, a major limitation of CRP as an early indicator of CAL is that elevated CRP levels can result from inflammation rather than signaling an underlying infection or complications such as CAL [11].

This has led to increased interest in other biomarkers, such as procalcitonin (PCT). PCT is primarily induced by bacterial components, though not exclusively [12,13]. Although PCT has shown potential as a negative test for CAL, the literature remains somewhat divided on its accuracy in diagnosing CAL after colorectal surgery [14-16].

In 2018, Tan and colleagues found that PCT serves as a valuable diagnostic tool for detecting intra-abdominal infections on postoperative day (POD) 5 after colorectal surgery and can assist in determining safe patient discharge [14]. In 2020, Su’a et al. suggested that PCT, especially on POD5, is a useful negative predictor for CAL after elective colorectal surgery [15]. However, they also highlighted that PCT alone is insufficient for detecting CAL. In 2022, Xu et al. reported that PCT levels on POD3 hold potential for early CAL diagnosis, with higher accuracy in patients undergoing laparoscopic surgery [16].

However, to our knowledge, no meta-analysis has specifically examined differences in PCT levels among colorectal cancer patients who do or do not develop CAL. Therefore, this meta-analysis aims to evaluate potential differences in PCT levels on PODs in colorectal cancer patients following colorectal surgery, with and without CAL.

## Review

Methods

Search Strategies of Studies

This research follows the "Preferred Reporting Items for Systematic Reviews and Meta-Analyses" guidelines [17]. We conducted an extensive database search on PubMed (2024/03/26), EMBL-EBI (European Molecular Biology Laboratory - European Bioinformatics Institute) (2024/03/26), and Scopus (2024/04/02) to find relevant studies without limiting the publication date. To ensure the identification of eligible studies, we carefully selected specific search terms, which were applied to the titles, abstracts, and keywords of the articles as follows: "(‘procalcitonin’ OR ‘PCT’) AND (‘colorectal’ OR ‘colorectal cancer’ OR ‘colorectal carcinoma’ OR ‘colorectal neoplasm’ OR ‘colorectal tumor’ OR ‘colon’ OR ‘colon cancer’ OR ‘colon carcinoma’ OR ‘colon neoplasm’ OR ‘colon tumor’ OR ‘rectal’ OR ‘rectal cancer’ OR ‘rectal carcinoma’ OR ‘rectal neoplasm’ OR ‘rectal tumor’) AND (‘anastomotic leakage’ OR ‘anastomotic leak’ OR ‘anastomosis leakage’ OR ‘anastomosis dehiscence’ OR ‘anastomotic dehiscence’)". We also conducted a manual search in the citing articles sections of PubMed and Scopus for eligible articles.

Inclusion and Exclusion Criteria for This Study

To be included, research had to be original, in English, and focus on circulating PCT levels in CAL after PODs of colorectal surgery in humans. While colorectal cancer patients were the main target, studies with benign condition patients were also considered if they included a control group. Studies in other languages, editorials, letters, retracted articles, reviews, non-human research, and those without a cancer patient group were excluded.

Selection of Studies

Two independent researchers carried out the article selection, quality assessment, and data extraction processes. Initially, they reviewed titles and abstracts to assess relevance. After this preliminary evaluation, they thoroughly examined the full texts of the selected studies to ensure they met the inclusion criteria. If disagreements arose, a third author was consulted to resolve conflicts through discussion and consensus.

Quality Assessment

Quality assessment of the included studies was performed using the "Quality Assessment of Diagnostic Accuracy Studies 2" tool, which evaluates both the quality and risk of bias in diagnostic accuracy studies [18].

Data Extraction

The following details were extracted from the articles: title, author names, publication year, country, study design, CAL definition, study groups, sample size, percentage of patients with CAL, type of sample collected, method used to measure PCT, and the corresponding results.

Statistical Analysis

This meta-analysis aimed to investigate the link between CAL and circulating PCT levels (ng/mL). To handle skewed data, we applied the method for unknown non-normal distributions (MLN) when studies reported medians instead of means and standard deviations (SD) ("https://smcgrath.shinyapps.io/estmeansd/") [19,20].

Meta-analyses were performed when at least three articles provided data, with PCT mean differences (MD) and 95% confidence intervals calculated. Heterogeneity was evaluated using the I² statistic, and a random-effects model (DerSimonian and Laird approach) was applied if I² exceeded 50% . Also, leave-one-out sensitivity analyses were performed. All calculations were performed in "OpenMeta [analyst]" [21].

Results

Search and Selection of Literature

**Figure 1:**
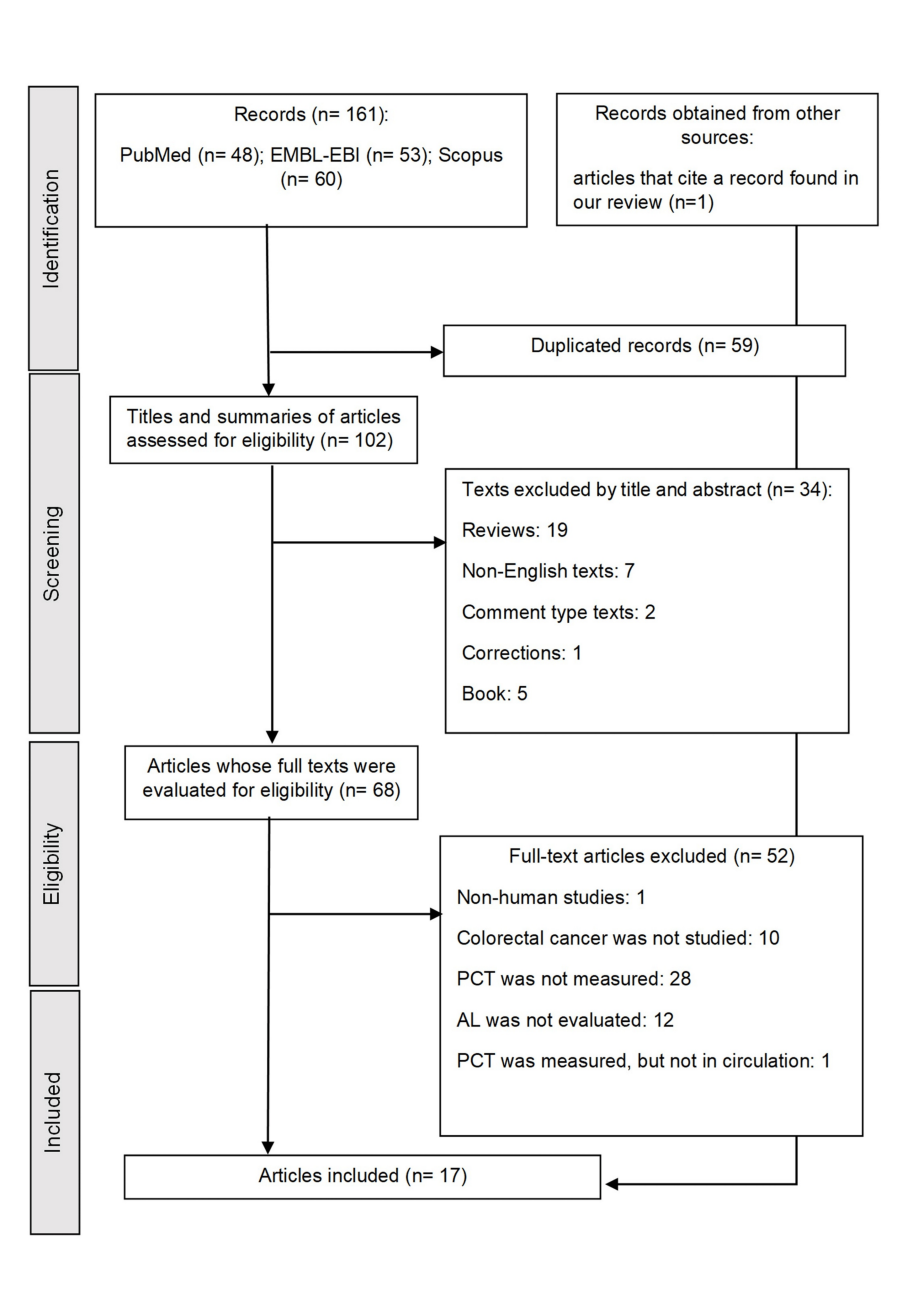
PRISMA chart. PRISMA: Preferred Reporting Items for Systematic Reviews and Meta-Analyses, EMBL-EBI: European Molecular Biology Laboratory - European Bioinformatics Institute, PCT: procalcitonin, AL: anastomotic leakage.

Figure 1 shows the literature search process. Figure 1 shows the literature search process following the PRISMA guidelines [17]. A total of 161 studies were initially identified through database searches. After removing 59 duplicate records, 102 studies were screened based on titles and abstracts, of which 34 were excluded for not meeting the inclusion criteria. Sixty-eight full-text articles were evaluated for eligibility, and 52 were excluded. One record was found through a manual search. Finally, 17 studies were included in the final analysis [22-38].

Table 1 provides a summary of the key characteristics of these studies, all published between 2012 and 2024. Of the selected studies, 13 were prospective in nature [22-28,32-37], two followed a case-control design [29,30], and two were retrospective studies [31,38].

**Table 1:** Characteristics of the included studies. CLIA: chemiluminescence immunoassay, CAL: colorectal anastomotic leakage, ELFA: enzyme-linked fluorescent assay, ELISA: enzyme-linked immunosorbent test, POD: postoperative day, QIF: quantitative immunofluorescence.

Study	Year of publication	Design	Country	Groups (n)	Method of PCT measurement	Blood derived sample	Monitoring (PODs)
Lagoutte et al. [22]	2012	Prospective	France	Non-CAL (87); CAL (13)	ELISA	Undefined	POD1 to POD4
Garcia-Granero et al. [23]	2013	Prospective	Spain	Non-CAL (188); CAL (17)	CLIA	Serum	POD1 to POD5
Giaccaglia et al. [24]	2014	Prospective	Italy	Non-CAL (72); CAL (9)	ELISA	Serum	POD1, POD3, POD5
Giaccaglia et al. [25]	2016	Prospective	Italy	Non-CAL (393); CAL (28)	ELISA	Serum	POD3, POD5
Zielińska-Borkowska et al. [26]	2016	Prospective	Poland	Non-CAL (141); CAL (16)	CLIA	Serum	POD1, POD5
Zawadzki et al. [27]	2016	Prospective	Poland	Non-CAL (50); CAL (5)	ELFA	Serum	POD1, POD3
Hayati et al. [28]	2017	Prospective	Malaysia	Non-CAL (67); CAL (3)	CLIA	Serum	POD3
Bilgin et al. [29]	2017	Case-control	Turkey	Non-CAL (43); CAL (7)	Undefined	Serum	POD1, POD3
Walker et al. [30]	2018	Case-control	Australia	Non-CAL (125); CAL (11)	Undefined	Undefined	POD1 to POD5
Chernyshov et al. [31]	2018	Retrospective	Russia	Non-CAL (89); CAL (11)	ELFA	Plasma	POD3, POD6
Muñoz et al. [32]	2018	Prospective	Spain	Non-CAL (128); CAL (6)	Undefined	Serum	POD1, POD2, POD3
Italian ColoRectal Anastomotic Leakage (iCral) Study Group [33]	2020	Prospective	Italy	Non-CAL (1470); CAL (76)	Undefined	Serum	POD2, POD3, POD6
Baeza-Murcia et al. [34]	2021	Prospective	Spain	Non-CAL (81); CAL (14)	Undefined	Undefined	POD3, POD5
El Zaher et al. [35]	2022	Prospective	Egypt	Non-CAL (183); CAL (22)	ELISA	Serum	POD1 to POD5
Janež et al. [36]	2022	Prospective	Slovenia	Non-CAL (40); CAL (3)	Undefined	Serum	POD1 to POD5
Rama et al. [37]	2022	Prospective	Portugal	Non-CAL (277); CAL (25)	CLIA and QIF	Serum and whole blood	POD1 to POD5
Hu et al. [38]	2024	Retrospective	China	Non-CAL (102); CAL (11)	Undefined	Serum	POD1, POD2, POD3

For the PCT determination (Table 1), four studies used chemiluminescence immunoassay (CLIA), another four employed enzyme-linked immunosorbent assay (ELISA), and two applied enzyme-linked fluorescent assays (ELFA). In seven studies, the method for PCT determination was not specified. As for the samples used to evaluate PCT, twelve studies focused on serum PCT levels, while only one measured PCT in plasma. One study assessed PCT in both serum and whole blood, and three studies did not specify the sample type for PCT measurement.

A total of 3,813 participants were included across the 17 studies, with 277 individuals diagnosed with CAL and 3,536 as control subjects. The main point of assessment for evaluating outcomes was POD3, which was analyzed in 16 studies focusing on PCT levels. Additionally, 12 studies measured PCT on POD1, nine on POD2, six on POD4, nine on POD5, and two on POD6 (Table 1).

Definitions of Colorectal Anastomotic Leakage

Table 2 presents the definitions of CAL utilized in the studies. Although the majority of studies emphasized clinical signs, they also incorporated imaging techniques. Nine studies used the Clavien-Dindo grading system to classify anastomotic leakage (AL) cases [39]. Five studies utilized the International Study Group of Rectal Cancer criteria for AL definition [40].

**Table 2:** Definition of anastomotic leakage in the studies. AL: anastomotic leakage, CT: computed tomography, POD: postoperative day, US: ultrasound.

Reference	Year	Definition
Lagoutte et al. [22]	2012	Postoperative peritonitis observed during reoperation, purulent or fecal wound drainage, and the presence of air or fluid collection in the anastomotic area on CT scans were noted. AL cases were classified as Clavien-Dindo grades (as major or minor).
Garcia-Granero et al. [23]	2013	Confirmed using an X-ray enema with hydrosoluble contrast in conjunction with a CT scan, endoscopy, or intraoperative examination. AL cases were classified as Clavien-Dindo grades (as major or minor).
Giaccaglia et al. [24]	2014	Presence of postoperative peritonitis identified during reoperation, fecaloid drainage, fecal material from the wound, contrast extravasation on enema, or the presence of air or fluid in the anastomotic region as visualized by a CT scan. AL cases were classified as Clavien-Dindo grades (as major or minor).
Giaccaglia et al. [25]	2016	In cases where AL was clinically suspected, a CT scan with a hydrosoluble contrast enema was performed. Then, AL was identified based on the presence of fecaloid drainage, fecal material coming from the wound, contrast leakage on an enema, signs of postoperative peritonitis observed during reoperation, or the detection of fluid or air in the anastomotic area on a CT scan. AL cases were classified as Clavien-Dindo grades (as major or minor).
Zielińska-Borkowska et al. [26]	2016	Postoperative peritonitis observed during reoperation, purulent or fecaloid drainage, and presence of air or fluid deposits in the anastomotic region on CT scan.
Zawadzki et al. [27]	2016	Defined only as “symptoms or signs of complication”. Complicated cases were classified as Clavien-Dindo grades.
Hayati et al. [28]	2017	Presence of peritonitis, frank pus, or fecal discharge from the drain, or by radiological evidence from a CT scan with contrast enema, or findings during surgery. AL cases were classified as Clavien-Dindo grades (as major or minor).
Bilgin et al. [29]	2017	Confirmed by a CT scan with rectal contrast enema, this assay was performed when clinical signs of AL were present. Indicators included fever after postoperative day 3, the presence of fecal matter or suspicious fluid from the drain, and abdominal tenderness.
Walker et al. [30]	2018	Evidence of luminal contrast extravasation on a CT scan or contrast enema, an abscess at the anastomosis site without contrast extravasation, a fistula originating from the anastomosis site, or an anastomotic defect identified during reoperation. AL cases were classified according to the International Study Group of Rectal Cancer system (A, B and C).
Chernyshov et al. [31]	2018	Presence of clear signs of peritonitis, enteric discharge from a drain or wound, clinical evidence of a rectovaginal fistula, and/or dehiscence of the anastomotic line identified through digital rectal examination or endoscopy. AL cases were classified according to the International Study Group of Rectal Cancer system (A, B and C).
Muñoz et al. [32]	2018	Observations during reoperation, fecaloid drainage, fecal material from the wound, contrast leakage on an enema, or the presence of air or fluid in the anastomotic area as seen on a CT scan. AL cases were classified as Clavien-Dindo grades (as major or minor).
Italian ColoRectal Anastomotic Leakage (iCral) Study Group [33]	2020	Any deviation from the expected postoperative recovery related to the anastomosis, such as pus or enteric fluid in drains, an abdominal or pelvic collection near the anastomosis observed on a CT scan, contrast leakage from the anastomosis during an enema, or anastomotic dehiscence found during reoperation for postoperative peritonitis. AL cases were classified as Clavien-Dindo grades (as major or minor).
Baeza-Murcia et al. [34]	2021	Patients exhibiting fever, prolonged ileus, physical signs of peritoneal irritation, or purulent/intestinal drainage through a drain underwent double or triple-contrast CT scans to confirm AL. AL cases were classified according to the International Study Group of Rectal Cancer system (A, B and C).
El Zaher et al. [35]	2022	AL was defined as the presence of evidence indicating a leak requiring active management, either through therapeutic or surgical intervention. This included findings such as discovery during reoperation, feculent drainage, fecal debris from the incision, contrast extravasation on an enema, or the presence of air or fluid in the anastomotic area detected by double contrast CT scan.
Janež et al. [36]	2022	Conditions exhibiting clinical or radiological signs consistent with AL, as defined by the International Study Group (ISG) criteria, which involve a defect in the intestinal wall at the anastomotic site—including suture and staple lines—leading to communication between the intra- and extraluminal compartments. AL cases were classified according to the International Study Group of Rectal Cancer system (A, B and C).
Rama et al. [37]	2022	In response to clinical deterioration and/or an increase in serum biomarkers, patients underwent additional imaging with abdominopelvic CT scan (and water-soluble contrast enema if there was a colorectal anastomosis). AL was defined by the following criteria: 1. Clinical criteria included enteric discharge from abdominal drains or wounds, rectovaginal fistula, or anastomotic defects identified during digital examination. 2. Radiological criteria (CT scan) involved extravasation of contrast administered endoluminally, intra-abdominal collections around the anastomosis, presacral abscesses adjacent to the anastomosis or perianastomotic air, and the presence of free intra-abdominal air. 3. Surgical findings during reoperation included anastomotic necrosis, signs of peritonitis, and identification of anastomotic defects. AL cases were classified as Clavien-Dindo grades (as major or minor) and the International Study Group of Rectal Cancer system (A, B and C).
Hu et al. [38]	2024	Presence of fecaloid discharge, fecal material coming from the wound, contrast leakage during an enema, signs of postoperative peritonitis noted during reintervention, or detection of fluid or air in the anastomotic region on a CT scan.

Evaluation of Risk of Bias and Applicability

The QUADAS-2 (Quality Assessment of Diagnostic Accuracy Studies-2) evaluation revealed that many of the included studies carried a significant risk of bias in patient selection, a finding further supported by the applicability assessment (Table 3).

**Table 3:** Quality Assessment of Diagnostic Accuracy Studies-2. Low risk: ✔, unclear/some concern: ?, high risk: X.

		Bias risk					Applicability		
Reference	Year of publication	Selection of patients	Index test		Reference standard	Flow and timing	Selection of patients	Index test	Reference standard
Lagoutte et al. [22]	2012	✔	?	✔		?	X	✔	✔
Garcia-Granero et al. [23]	2013	?	✔	✔		✔	✔	✔	✔
Giaccaglia et al. [24]	2014	✔	✔	✔		?	X	✔	✔
Giaccaglia et al. [25]	2016	✔	✔	✔		✔	✔	✔	✔
Zielińska-Borkowska et al. [26]	2016	X	✔	✔		?	X	✔	✔
Zawadzki et al. [27]	2016	X	✔	✔		✔	X	✔	✔
Hayati et al. [28]	2017	X	✔	✔		✔	X	✔	✔
Bilgin et al. [29]	2017	X	?	✔		✔	X	✔	✔
Walker et al. [30]	2018	X	?	✔		✔	X	✔	✔
Chernyshov et al. [31]	2018	?	✔	✔		?	?	✔	✔
Muñoz et al. [32]	2018	✔	?	✔		?	X	✔	✔
Italian ColoRectal Anastomotic Leakage (iCral) Study Group [33]	2020	?	?	✔		✔	✔	✔	✔
Baeza-Murcia et al. [34]	2021	✔	?	✔		✔	X	✔	✔
El Zaher et al. [35]	2022	?	✔	✔		?	X	✔	✔
Janež et al. [36]	2022	?	?	✔		?	X	✔	✔
Rama et al. [37]	2022	X	✔	✔		?	?	✔	✔
Hu et al. [38]	2024	?	?	✔		?	X	✔	✔

Findings on PCT in the Studies

A summary of the findings from studies analyzing PCT levels is provided in Table 4. Since Lagoutte et al. found elevated PCT levels in CAL patients from POD1 to POD4 following surgeries for colorectal cancer, several studies have attempted to confirm these results [22]. Although inconsistent findings are reported for PCT levels across most PODs evaluated in the studies, POD3 stands out as the most consistent and extensively studied (16 reports), with 12 studies reporting elevated PCT levels on POD3 in CAL patients compared to those without CAL.

**Table 4:** Results regarding PCT in the included studies. PCT: procalcitonin, AL: anastomotic leak, NA: not applicable, POD: postoperative day, (↑): significantly increased, (-): non-differences.

Reference	PCT results in the PODs
	Serum/plasma/whole blood
Lagoutte et al., 2012 [22]	1↑,2↑,3↑,4↑
Garcia-Granero et al., 2013 [23]	1-,2-,3↑,4↑,5↑
Giaccaglia et al., 2014 [24]	1-,3↑,5↑
Giaccaglia et al., 2016 [25]	3↑,5↑
Zielińska-Borkowska et al., 2016 [26]	1↑,5↑
Zawadzki et al., 2016 [27]	1-,3↑
Hayati et al., 2017 [28]	3↑
Bilgin et al., 2017 [29]	1-,3↑
Walker et al., 2018 [30]	1-,2-,3-,4-,5-
Chernyshov et al., 2018 [31]	3↑,6↑
Muñoz et al., 2018 [32]	1↑,2↑,3↑
Italian ColoRectal Anastomotic Leakage (iCral) Study Group, 2020 [33]	2↑,3↑,6↑
Baeza-Murcia et al., 2021 [34]	3-,5-
El Zaher et al., 2022 [35]	1↑,2↑,3↑,4↑,5↑
Janež et al., 2022 [36]	1-,2-,3-,4-,5-
Rama et al., 2022 [37]	1-,2-,3-,4-,5-
Hu et al., 2024 [38]	1-,2-,3↑

Meta-Analysis of PCT Concentrations

Several studies were excluded from the statistical analysis in this meta-analysis due to inconsistencies in data reporting. For example, in the studies by Lagoutte et al. [22], Giaccaglia et al. [24,25], Walker et al. [30], and Chernyshov et al. [31], data dispersion measures were not provided in the text, tables, or figures, which restricted access to this information. Furthermore, one study eligible for the meta-analysis reported their PCT results using median, Q1, and Q3 values [26]. To address this, we applied the Cai et al. method to estimate the mean and standard deviation [19].

**Figure 2:**
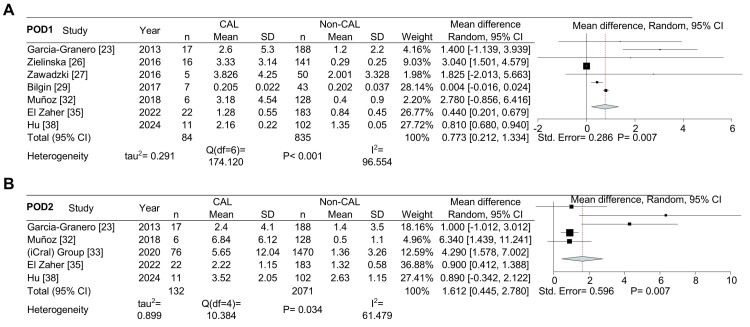
Forest plot showing the mean difference in procalcitonin levels (ng/mL) between patients with colorectal anastomotic leakage (CAL) and those without CAL on POD1 and POD2. Mean difference in procalcitonin levels for postoperative days (POD) 1 (A) and POD2 (B).

**Figure 3:**
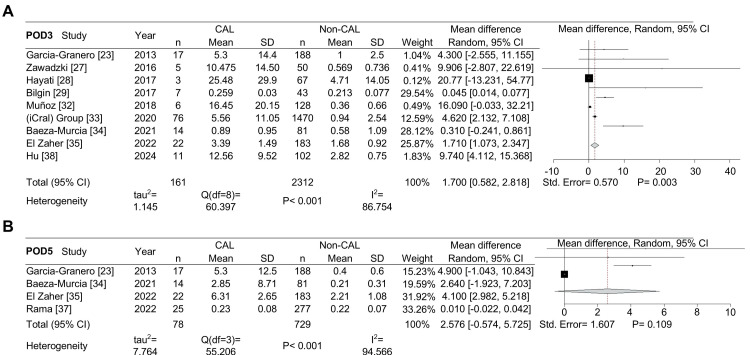
Forest plot showing the mean difference in procalcitonin levels (ng/mL) between patients with colorectal anastomotic leakage (CAL) and those without CAL on POD3 and POD5. Mean difference in procalcitonin levels for postoperative days (POD) 3 (A) and POD5 (B).

In the statistical analysis, we initially included seven studies for POD1 (Figure 2A), which decreased to five for POD2 (Figure 2B). Nine studies were available for POD3 analysis (Figure 3A), and data from four studies were used for POD5 (Figure 3B). The combined data showed that PCT levels (in ng/mL) were significantly higher in CAL patients compared to non-CAL patients on POD1 (MD: 0.773, 95% CI: 0.212-1.334, P = 0.007), POD2 (MD: 1.612, 95% CI: 0.445-2.780, P = 0.007), and POD3 (MD: 1.700, 95% CI: 0.582-2.818, P = 0.003). However, no significant difference was observed on POD5 (P = 0.109).

**Figure 4:**
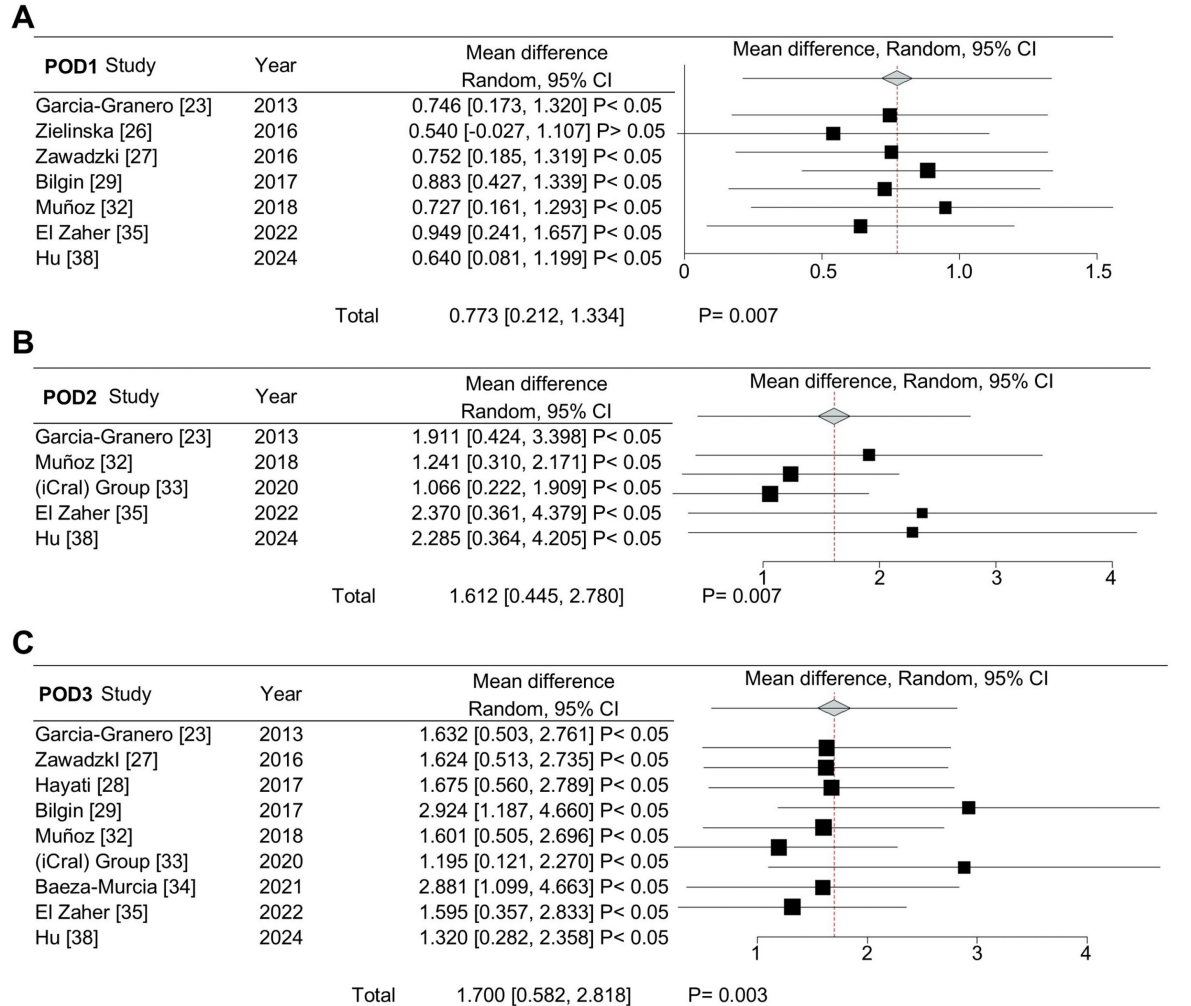
A leave-one-out sensitivity analysis was performed on the mean procalcitonin (ng/mL) levels of colorectal anastomotic leakage (CAL) and non-CAL patients. Mean difference in procalcitonin levels for postoperative days (POD) 1 (A), POD2 (B), and POD3 (C).

A leave-one-out sensitivity analysis was conducted to validate the findings for POD1, POD2, and POD3 (Figure 4), in order to address the high heterogeneity observed in the meta-analysis. The results indicated that the study by Zielinska-Borkowska et al. [26] had a substantial impact on the differences noted for POD1 (Figure 4A). When this study was excluded from the pooled PCT data, the differences between CAL and non-CAL patients ceased to be statistically significant. However, PCT levels remained significantly higher in CAL patients compared to non-CAL patients on POD2 (Figure 4B) and POD3 (Figure 4C).

**Figure 5:**
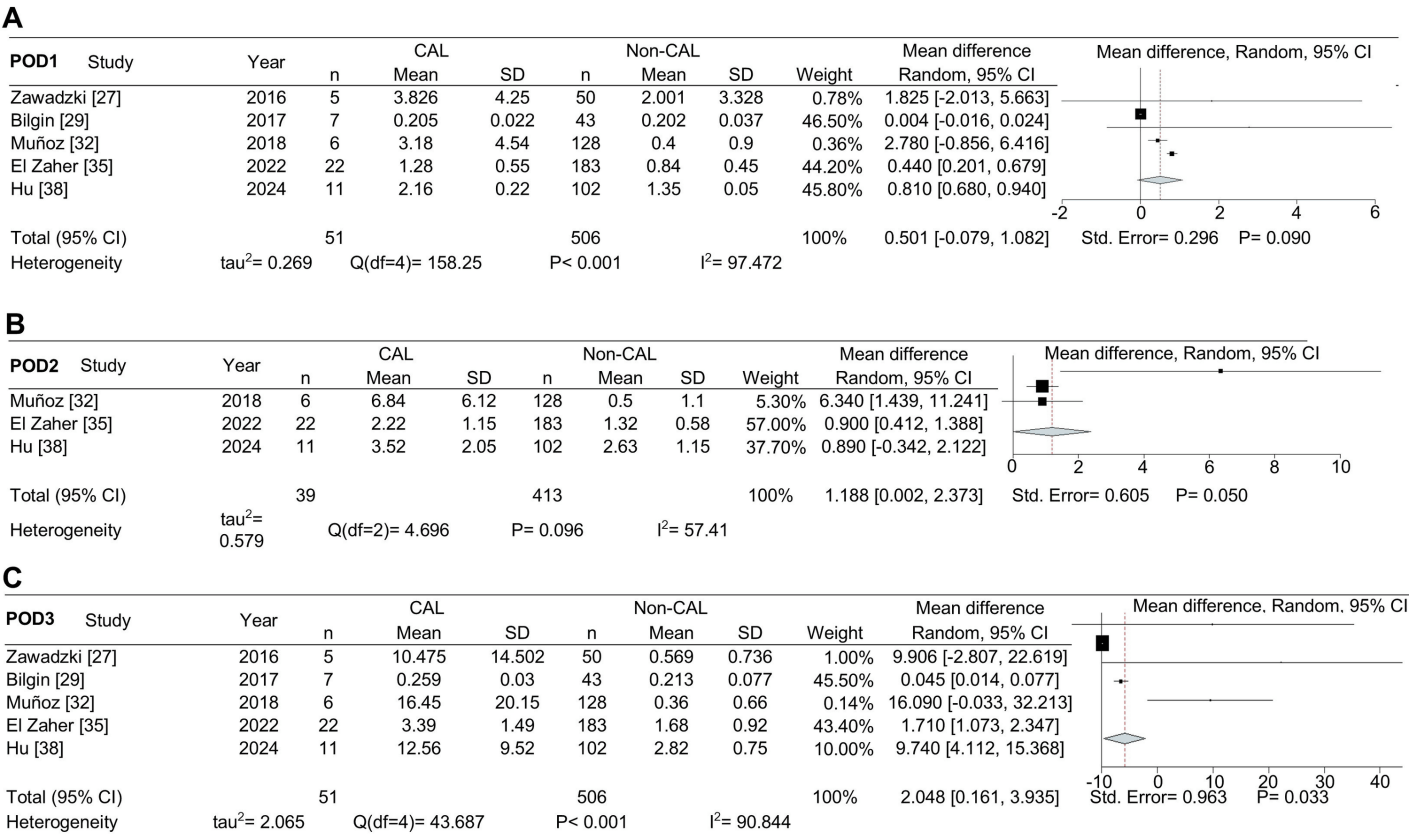
Forest plot showing the mean difference in procalcitonin (ng/mL) between colorectal anastomotic leakage (CAL) and non-CAL patients in studies exclusively involving cancer patients. Mean difference in procalcitonin levels for postoperative days (POD) 1 (A), POD2 (B), and POD3 (C).

Although all studies included colorectal cancer patients, some also included patients with benign conditions. To ensure an analysis focused solely on colorectal cancer patients, we selected studies that exclusively involved these patients and conducted a meta-analysis to compare PCT differences between CAL and non-CAL colorectal cancer patients. Regarding this statistical analysis, we included five studies for POD1 and POD3 (Figure 5A, 5C) and three studies for POD2 (Figure 5B). The combined data from studies that focused solely on cancer patients indicated that PCT levels were higher in CAL patients on POD2 (MD: 1.188, 95% CI: 0.002-2.373, P = 0.050) and POD3 (MD: 2.048, 95% CI: 0.161-3.935, P = 0.033) compared to non-CAL patients. No statistically significant differences were detected when CAL and non-CAL patients were compared for PCT levels on POD1 (P = 0.090).

**Figure 6:**
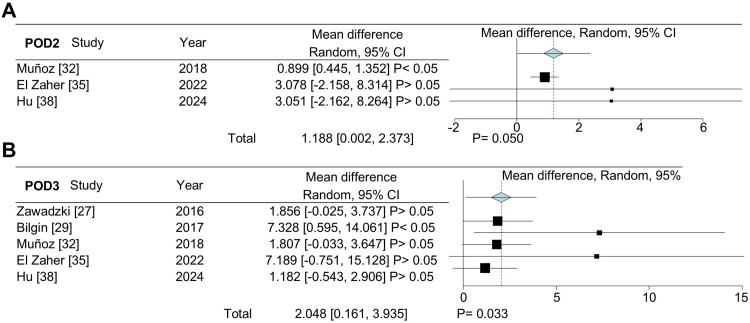
Sensitivity analysis using the leave-one-out method on the mean procalcitonin (ng/mL) levels of colorectal anastomotic leakage (CAL) and non-CAL patients in studies exclusively involving cancer patients. Mean difference in procalcitonin levels for postoperative days (POD) 2 (A), and POD3 (B).

Once again, to address the anticipated high heterogeneity, we performed a leave-one-out sensitivity analysis to confirm our findings for POD2 and POD3, focusing on PCT levels in the studies that exclusively included colorectal cancer patients (Figure 6). Studies by El Zaher et al. [35] and Hu et al. [38] significantly influenced the differences observed in POD2 (Figure 6A). When either of these studies was removed from the analysis on PCT levels, the differences between patients with and without CAL were no longer significant. Similarly, excluding studies from the POD3 analysis rendered the differences between CAL and non-CAL patients insignificant (Figure 6B).

Discussion

PCT levels have been suggested as reliable markers of CAL. Therefore, we expected to find significant differences in PCT levels when comparing pooled data. Based on our meta-analysis, PCT levels are significantly different between CAL and non-CAL patients on POD2 and POD3. This shows that POD2 and POD3 PCT levels could be a potentially reliable marker of CAL following colorectal surgery. However, when considering only studies that exclusively included colorectal cancer patients, we found that the significant difference was no longer present. One explanation is that the number of studies exclusively including colorectal cancer patients in our meta-analysis is small-only three studies for POD2 and five for POD3. Therefore, more studies are needed to investigate differences in PCT levels between colorectal cancer patients with and without CAL.

In healthy individuals, PCT is synthesized by the thyroid C-cells but this PCT does not enter the bloodstream. Consequently, the PCT concentration in healthy individuals remains very low (approximately 0.05 ng/mL). However, research has indicated the existence of alternative PCT release mechanisms related to infections and inflammatory responses that function independently of the typical pathway [41,42].

During inflammation, localized bacterial infections, and sepsis, which are common occurrences in the PODs following colorectal surgery, especially in cases of CAL-PCT production may be triggered by bacterial lipopolysaccharides (LPS), endotoxins, or inflammatory mediators such as IL-1β, IL-2, IL-6, and TNF-α [41-43]. Studies have shown that in the presence of bacterial infections or systemic inflammation, PCT levels start to rise within 2-4 hours, peaking at 24-48 hours, with a half-life of approximately 24 hours thereafter [16,42]. As a result, PCT holds potential as a biomarker for identifying complications like CAL after colorectal surgery.

Given that PCT has been suggested as a biomarker for infection and sepsis, several meta-analyses have been conducted to evaluate its potential as a diagnostic tool for detecting CAL [14-16]. In 2020, Su’a et al. found that PCT serves as a valuable negative predictor for CAL on POD5 [15]. However, early CAL typically occurs within the first five days following colorectal surgery [7,15,44]. Furthermore, some authors have noted that in institutions with enhanced recovery protocols, such as the enhanced recovery after surgery (ERAS) protocol, waiting for inflammatory marker measurements until POD5 may not be practical [14]. More recently, Xu et al. demonstrated that PCT levels on POD3 show potential for early CAL diagnosis, with higher diagnostic accuracy in patients undergoing laparoscopic surgery compared to open procedures [16]. They also recommended a cutoff range of 0.7-1.3 ng/mL for accurate diagnosis and safe discharge [16].

Here, we confirm that PCT levels measured on POD3 could serve as effective biomarkers for detecting CAL. While we observed significant differences in PCT levels on POD3 when comparing patients with and without CAL after colorectal surgery, these findings no longer held when we limited the analysis to studies focusing exclusively on colorectal cancer patients who either developed or did not develop CAL at any POD. Although our results are consistent with those reported in two earlier meta-analyses [15,16], those studies did not specifically examine PCT levels in colorectal cancer patients with or without CAL. To our knowledge, this is the first meta-analysis to assess PCT levels solely in colorectal cancer patients, excluding participants with benign conditions.

The search for reliable biomarkers for CAL is ongoing. Some authors have suggested that PCT would be more effective when used alongside other clinical data and biomarkers, such as CRP [38,45], but further studies are needed to fully assess its potential in diagnosing CAL. Given the high morbidity associated with CAL in colorectal cancer patients undergoing surgery, early detection and prompt intervention are critical. Our results highlight the need to consider the specific profile of colorectal cancer patients when evaluating biomarkers like PCT, as their postoperative course differs from those with benign conditions [46].

Increased PCT levels have been observed in patients with colorectal cancer. In 2013, Keramidaris and colleagues found that half of their cancer patient cohort had elevated PCT levels, with the highest levels observed in those with metastases. However, they did not establish a link between elevated PCT and an increase in postoperative complications (which were not specified but were presumed to be septic in nature) [47]. In a 2021 study, Miyake et al. also reported high PCT levels in preoperative colorectal cancer patients and identified an association between elevated PCT and poorer overall survival in those undergoing surgery for colorectal cancer [48].

In the study by Keramidaris et al., they examined bacterial translocation (BT) in colorectal cancer patients, a condition that is common in this group [47,49]. BT occurs when live bacteria or their byproducts escape from the intestinal tract into extra-luminal areas. Keramidaris and colleagues cited that in colorectal cancer patients, this phenomenon can be attributed to increased gut permeability due to the disruption of bowel structure by malignant tissues [47,50]. Additional factors influencing the degree of bacterial translocation include changes in gut flora and suppressed immune responses [47]. Consequently, BT could account for the elevated PCT levels observed in these patients.

Additionally, since inflammatory mediators such as IL-1β, IL-2, IL-6, and TNF-α have been suggested to contribute to elevated PCT levels [41-43], it is likely that the inflammation occurring during tumorigenesis and cancer progression in colorectal cancer patients also contributes to their elevated PCT levels. It is known that colorectal cancer patients exhibit dysregulation in the levels of numerous cytokines, including all the aforementioned molecules [51]. In 2015, Chaftari et al., studying various types of cancer, including colorectal cancer, reported elevated levels of PCT and IL-6 in their cancer patients compared to controls, and suggested that PCT has potential for detecting cancer progression and predicting bacteremia in cancer patients [52]. Therefore, it would also be important for future studies to consider the possibility that elevated levels of inflammatory cytokines in colorectal cancer patients could contribute to their elevated PCT levels.

Overall, it is likely that the lack of differences in mean PCT levels observed in our study specifically in colorectal cancer patients is due to the fact that these patients often have elevated PCT levels even in the preoperative period. Additionally, their recovery process and response to surgery tend to differ from other patients, such as those with benign conditions. Further research is necessary to better understand this PCT response, and special consideration should be given to its use as a biomarker for CAL diagnosis in colorectal cancer patients after surgery [47]. To address this, studies with longitudinal designs are needed, ideally involving colorectal cancer patients prior to surgery. Also, it would be valuable to observe the behavior of PCT levels across different populations. To this end, studies should be conducted in various geographical regions, especially in those where this analysis has not yet been performed. Finally, as PCT has been suggested to be a more effective diagnostic tool when combined with other analytes, such as CRP and inflamatory markers, it is important to conduct studies that explore such combined diagnostic approaches.

Strengths and limitations

This is the first meta-analysis aimed at differentiating PCT levels in CAL patients compared to non-CAL patients, exclusively focusing on patients with colorectal cancer. Virtually all eligible studies to date were included, with no time restrictions, in accordance with international PRISMA (Preferred Reporting Items for Systematic Reviews and Meta-Analyses) guidelines. Lastly, in addition to including studies that reported results with means and standard deviations (SD), the meta-analysis also considered studies that presented data with medians and quartiles, which were converted to means and SD, to include a greater number of studies and patients in our analysis.

Limitations

First, meta-analysis is founded on a limited number of studies included in the analysis. Second, the variability in PCT results across different articles led to significant heterogeneity in our meta-analysis. Third, none of the included studies were conducted in the Americas, introducing potential selection bias and limiting the generalizability of the findings to this region. Fourth, it is important to recognize that the selection of methods can influence the estimates in the analysis, as different approaches may be used to calculate the mean and standard deviation.

## Conclusions

The meta-analysis confirms that PCT levels have potential as early markers for CAL after colorectal surgery when all types of patients are included in the analysis. However, PCT levels were no longer significantly different between patients with and without CAL when only patients with colorectal cancer were considered. Consequently, the existing evidence suggests that PCT levels should not be considered as early markers for CAL following colorectal surgery, particularly in colorectal cancer patients. Further research is necessary to evaluate whether PCT could be a reliable biomarker for CAL in this context.
